# Long Non-Coding RNAs as New Biomarkers in Lupus Nephritis: A Connection Between Present and Future

**DOI:** 10.7759/cureus.9003

**Published:** 2020-07-05

**Authors:** Galya Mihaylova, Vasil Vasilev, Mariya B Kosturkova, George S Stoyanov, Maria Radanova

**Affiliations:** 1 Biochemistry, Molecular Medicine and Nutrigenomics, Medical University of Varna, Varna, BGR; 2 Nephrology, "Tsaritsa Yoanna – ISUL” University Hospital, Sofia, BGR; 3 Propaedeutics of Internal Diseases, Medical University of Varna, Varna, BGR; 4 General and Clinical Pathology/Forensic Medicine and Deontology, Medical University of Varna, Varna, BGR

**Keywords:** systemic lupus erythematosus, lupus nephritis, biomarkers, lncrna

## Abstract

Lupus nephritis (LN) is a severe complication of systemic lupus erythematosus (SLE). LN often leads to kidney failure, affecting the quality of a patient's life. There are several classical biomarkers that assist nephrologists’ daily practice. For more than 50 years, anti-double stranded DNA antibodies and complement components C3 and C4 have been used for LN disease activity evaluation. The major obstacle in the usage of conventional biomarkers is that none of them have both high specificity and high sensitivity. Moreover, an invasive kidney biopsy is still the gold standard for renal involvement detection in SLE patients. Therefore, new non-invasive biomarkers are needed for the early and accurate establishment of LN. Among the promising candidates are long non-coding RNAs (lncRNAs). Their dysregulation appears to have predictive and diagnostic potential. Furthermore, these biomarkers like other conventional biomarkers give insight into the pathogenesis of LN. This review aims to summarize the available information on lncRNAs in SLE patients and to present their future opportunities to add to the conventional biomarkers in the diagnosis and monitoring of LN.

## Introduction and background

Systemic lupus erythematosus (SLE) is an autoimmune disease that mainly affects women of childbearing age. Etiological factors of the disease are hormonal and immunological influences, as well as genetic predisposition [1]. The disease may affect many organs such as the skin, joints, lungs, heart, and central nervous system, as well as the kidneys, damage to which is especially associated with poor prognosis [2]. Thus, lupus nephritis (LN) is one of the most severe complications of SLE and is present in about 60% of patients [3]. The pathophysiological findings in patients with active LN include deposition of immune complexes in the mesangium and/or the subendothelial space, with clinical manifestations ranging from microscopic proteinuria to nephrotic syndrome, erythrocyturia, leukocyturia, thrombocytopenia, anemia, high titers of anti-double stranded (anti-ds) DNA and anti-C1q antibodies, and reduced levels of complement components C3 and C4 [4].

Treatment depends on the severity of the disease, but it may be a lifelong process often associated with numerous treatment-related complications for the patients and is a substantial financial burden, as well as decreasing both the patients' and their families' quality of life.

A kidney biopsy is the gold standard to confirm the diagnosis and as a method can determine the severity of LN. Some non-invasive serum biomarkers are considered stable and repeatable; however, they are generally used for patient monitoring. For example, conventional immunological serum biomarkers help clinicians in confirming the presence of the disease or in the prognosis of clinical events such as flares in patients. Regardless of their proven role in LN follow-up, the immunological biomarkers do not display enough specificity and/or sensitivity alone [2,3].

The use of a single standard immunological biomarker often is not enough for the clinical decision. Besides, a low prevalence of biomarkers in LN patients may affect their clinical application. These facts identify the need for further study on novel biomarkers. Finding more informative biomarkers with relatively high specificity and sensitivity for monitoring of LN is essential for early detection of renal involvement and appropriate treatment.

In recent years, the use of more advanced screening technologies such as gene expression, microarray technology, and deep sequencing have opened new categories of biomarkers such as non-protein encoding RNAs circulating in the blood. Non-coding RNAs such as long non-coding RNAs (lncRNAs) have been reported to play a role in autoimmune diseases [5]. Their presence in plasma and serum makes them potential non-invasive biomarkers for disease activity and progression [6].

This study aims to make a brief overview of the new generation of biomarkers such as lncRNAs found in LN and to evaluate their potential role as diagnostic and prognostic tools for the disease alone or in combination with the conventional immunological markers for activity.

## Review

Commonly used serological immunological markers for evaluation of the activity of LN

Laboratory values such as plasma creatinine, C3, C4, anti-dsDNA and anti-C1q antibodies, proteinuria, and hematuria are classical clinical and diagnostic biomarkers for LN. The measurement of plasma levels of antinuclear antibodies (ANAs), anti-dsDNA, anti-C1q antibodies, C3, and C4 are utilized for the diagnosis and evaluation of the immunological activity of LN. Their clinical utility to detect LN flares is characterized by variable sensitivity and/or specificity (Table 1).

**Table 1:** Immunological biomarkers in lupus nephritis *No value in the disease follow-up. LN, lupus nephritis; ANA, anti-nuclear antibodies; anti-dsDNA, anti-double stranded DNA; anti-C1q, complement component 1q; C3, complement component 3; C4, complement component 4

Biomarkers	Prevalence in LN, %	Sensitivity/specificity, %	Clinical utility
ANA*	97.4	95–100/low specificity	Positive whatever disease activity
Anti-dsDNA	63.3	70–90/49–97.7	Correlate with the presence of LN and disease activity
Anti-C1q	Up to 97	13.33–100/39.05–97.58	Predict LN flares
C3	68–84.6	64.1–70/73–88.4	Poor clinical utility
C4	74–87.2	49–51.3/74–95.3	Poor clinical utility

ANAs are detected in more than 90% of SLE patients who have not undergone immunosuppressive treatment [4]. They are characterized by high sensitivity but low specificity in SLE. Therefore, ANAs have a low positive predictive value for SLE, especially if they are in low titers [7]. There are no data regarding a strong correlation between ANA levels and the development of LN, as well as its severity [8].

In comparison with ANAs, anti-dsDNA antibodies have poor sensitivity but higher specificity [9]. According to several studies, anti-dsDNA strongly correlates with the presence and clinical activity of LN [10,11]. The increase in anti-dsDNA levels precedes exacerbations of the disease [12,13]. In contrast, some other studies report that not all patients with anti-dsDNA develop LN [14] or that anti-dsDNA does not correlate with LN or its flares [15]. Regardless, follow-up serum levels of anti-dsDNA in LN patients may be informative since there is a relation between anti-dsDNA and disease activity according to predominant publications.

A superior specificity over anti-dsDNA for renal flares is shown by anti-C1q [16-18]. High anti-C1q levels present in the serums of approximately 20% to 50% of the SLE patients [19,20]. The positive predictive value of anti-C1q antibodies for LN is determined by some authors to be about 58% and as a negative predictive value for LN (between 91% and 100%) [17,21,22]. An increase in their titters is observed to have a predictive value for the development of exacerbation of LN and LN recurrence, and it is associated with proliferative nephritis classes [21,23-27].

In recent years, two anti-complement autoantibodies have become clinically relevant for patients with LN. Anti-C3 antibodies recently characterized by our team are detected in up to 30% of the patients with LN [28]. Our data show that levels of anti-C3 correlate with the disease severity and can be used to identify patients with LN who were prone to flare [28,29]. Other new anti-complement autoantibodies in LN patients with a frequency of 22.5% are antibodies against properdin - the only positive regulator of the complement system. In a previous study, we have established that higher levels of anti-properdin antibodies are associated with high levels of ANAs, anti-dsDNA, low levels of C3 and C4, and certain histological signs of LN activity. Anti-properdin antibodies and anti-dsDNA in combination have a high negative predictive value for patients with severe LN [30]. Anti-C3 and anti-properdin antibodies are new potential biomarkers in LN, which still need to be validated in multiple, large independent cohorts.

The decreased plasma levels of C3 and C4 in LN patients are associated with poor prognosis. Nevertheless, alone they have a poor clinical utility to predict SLE renal flare [31]. The plasma levels of C3 and C4 decrease before the activation of LN, and their multiple follow-ups have a higher diagnostic and prognostic significance for the condition in comparison with a single measurement of C3 and C4 [32,33].

All these classic immunological biomarkers for LN activity have some limitations concerning their role to identify renal damage when they are tested independently; however, their specificity increases when they are combined.

LncRNAs as new biomarkers in SLE

lncRNAs are non-protein-coding RNAs containing more than 200 nucleotides that regulate a wide spectrum of biological processes through a variety of mechanisms. LncRNAs play a role in the pathogenesis of malignant, metabolic, and autoimmune diseases. The role of lncRNAs in malignancies is relatively well established; however, the significance of lncRNAs in chronic inflammatory diseases is still poorly understood [5]. Recently, it has been established that changes in lncRNAs expression have a direct role in the initiation and progression of SLE and may be responsible for organ injury in different sites, the form of clinical manifestations, and changes in the established markers of disease activity and progression [34]. Over the last few years, several studies have reported that some lncRNAs have a different expression in SLE patients with and without LN.

Evaluating the circulating lncRNAs in SLE has been considered as a promising non-invasive set of biomarkers for early identification and prognosis of the condition. Currently, several lncRNAs are investigated as biomarkers in SLE [6]. Some of them have a diagnostic role, and others could serve to distinguish SLE patients with LN from those without.

Many of these lncRNAs have been studied for their relationships with clinical parameters of SLE, which could help to identify SLE patients with severe disease and as a tool to monitor disease progression [5]. Less is known about how their different expressions critically affect the pathological pathways in SLE.

LncRNAs that may specifically identify SLE patients

This group presents lncRNAs, which have different expression in SLE patients but are not investigated in patients with LN. Their regulation and association with some clinical markers of the disease are shown in Table 2.

**Table 2:** Long non-coding RNAs detected in SLE patients ↑, upregulated; ↓, downregulated; (+), positive correlation; (-), negative correlation; SLE, systemic lupus erythematosus; PBMCs, peripheral blood mononuclear cells; SLEDAI, systemic lupus erythematosus disease activity index; ESR, erythrocyte sedimentation rate; CRP, C-reactive protein; anti-dsDNA, anti-double stranded DNA; LN, lupus nephritis; eGFR, estimated glomerular filtration rate; IL, interleukin; SIRT1, silent information regulator 1; IFN, interferon

Long non-coding RNA	Regulation	Used material of SLE patients	Correlation with clinical parameters of disease activity	Reported function or potential clinical utilization
GAS5	↓ in SLE	Plasma, PBMCs	SLEDAI (-) ESR (-) CRP (-)	A diagnostic marker for SLE
NEAT1	↑ in SLE	PBMCs	SLEDAI (+)	Regulate the expression of inflammatory chemokines and cytokines Assessment of disease activity
CircIBTK	↓ in SLE	PBMCs	SLEDAI (+) anti-dsDNA (+) C3 (-)	Regulate DNA demethylation and AKT pathway by sponging miR-29b Assessment of disease activity
ENST00000448942	↓ in SLE	CD3^+^ T cells	ESR (+) anti-Sm antibodies (+)	-
uc001ykl.1	↓ in SLE	CD3^+^ T cells	ESR (+) CRP (+)	-
TSIX	↑ in SLE	PBMCs	SLEDAI (+)	Assessment of disease activity
lnc3643	↓ in SLE	PBMCs	ESR (-) CRP (-)	-
lnc7514	↓ in SLE	PBMCs	ESR (-) SLEDAI (-)	-
lnc0597	↓ in SLE ↓ in LN ↑ in SLE ↑ in LN	PBMCs, plasma	SLEDAI (-) C3 (-)	A diagnostic marker for SLE Distinguish SLE with LN from SLE without LN
lnc-DC	↓ in SLE ↑ in LN ↑ in SLE ↑ in LN	Plasma	C3 (-) CRP (-) SLEDAI (+) eGFR (-)	Distinguish SLE with LN from SLE without LN Assessment of disease activity
linc0949	↓ in SLE ↓ in LN	PBMCs, plasma	SLEDAI (-) C3 (+)	A diagnostic marker for SLE Assessment of disease activity
MALAT-1	↑ in SLE ↑ in LN	Plasma, PBMCs	eGFR (-) anti-dsDNA (-) creatinine (+)	Regulates SIRT1 pathway and effect on the expression of IL-21
CTC-471J1.2	↓ in SLE ↓ in LN	Plasma	SLEDAI (-) eGFR (+)	A diagnostic marker for LN
TUG1	↓ in SLE ↓ in LN	PBMCs	SLEDAI (-) ESR (-) proteinuria (-) C3 (+)	Distinguish SLE with LN from SLE without LN Assessment of disease activity Regulate of miR-233 and SIRT1
circHLA-C	↑ in LN	kidney tissue	Proteinuria (+) serum creatinine (+) renal activity index (+) crescentic glomeruli (+)	Sponge miR-150
circRNA-002453	↑ in LN	Plasma	renal SLEDAI (+) proteinuria (+)	A diagnostic marker for LN Distinguish SLE with LN from SLE without LN
RP11-2B6.2	↑ in LN	Kidney tissue, PBMCs	proteinuria (+) IFN-I score (+)	A diagnostic marker for LN Regulate IFN-I signaling pathway

LncRNA growth arrest-specific transcript 5 (GAS5) is aberrantly expressed in SLE patients when compared with healthy controls [35-38]. GAS5 levels are significantly lower in patients with active SLE and are negatively correlated with the SLE disease activity index (SLEDAI) score, erythrocyte sedimentation rate (ESR), and C-reactive protein (CRP) [35,36]. The CD4+ T cells of SLE patients with ulcerations have a high expression of GAS5 [38]. Since GAS5 is overexpressed in an immune cell, probably it has specific functions in autoimmune diseases such as SLE. GAS5 and linc0597 in combination with lnc0640, lnc5150, and lnc7074 as a panel of lncRNAs could distinguish SLE from other rheumatic diseases such as rheumatic arthritis and Sjögren’s syndrome [37].

Nuclear enriched abundant transcript 1 (NEAT1), also known as ENST00000501122.2, is upregulated in SLE and shows a positive correlation with SLEDAI-2K score [39,40] and with inflammatory cytokines and chemokines (IL, interleukin-6 [IL-6] and CXCL10), involved in the pathology of SLE [39]. Probably NEAT1 has a major function in SLE flares and it may be a useful marker in the monitoring of SLE activity.

lncRNA hsa_- circ_0077179, which is derived from the IBTK (Inhibitor of Bruton tyrosine kinase) gene locus and also termed circIBTK, is downregulated in SLE patients [41]. Levels of circIBTK correlate with SLEDAI-2K score, anti-dsDNA, and C3 level in SLE patients [41].

The lncRNAs ENST00000448942 and uc001ykl.1 are also downregulated in SLE patients [42]. uc001ykl.1 correlates with CRP and ENST00000448942, with levels of anti-Smith antibodies as an expression of both lncRNAs correlates with ESR [42].

LncRNA antisense for X-inactive-specific transcript, ENST00000604411.1 (TSIX), is overexpressed in SLE patients and positively correlated with the SLEDAI score [40]. Wang et al. also report an aberrant expression of two others lncRNAs, lnc-HSFY2-3:3 and lnc-SERPINB9-1:2, which still have unknown function [40].

Lnc5150 has potential as a diagnostic marker for SLE [43]. Levels of lnc3643 are decreased in SLE patients with proteinuria in comparison to SLE patients without proteinuria and correlate with CRP and ESR [43]. Lnc7514 levels are low in SLE patients positive for anti-dsDNA and correlate with ESR and SLEDAI-2K [43].

It would be interesting to follow-up on the levels of these lncRNAs in LN patients in the context of clinical markers of disease activity.

LncRNAs that may distinguish SLE patients with LN from SLE patients without LN

This group includes relatively more investigated lncRNAs in SLE. Regulation and association of these lncRNAs with some clinical markers of disease activity are presented in Table 2.

Lnc0597 has significantly increased expression in SLE patients with hypocomplementemia [35] but is downregulated in SLE patients with proteinuria and negatively correlates with SLEDAI-2K score [36]. Linc0597 could serve as a diagnostic biomarker for SLE patients [35-37,44] and also for distinguishing LN from SLE without LN [36].

LncRNA found in dendritic cells (lnc-DC) is downregulated in SLE patients in comparison with healthy controls [35-36] and negatively correlates with C3 [35], CRP levels, and duration of disease [36]. In the group of SLE patients, lnc-DC is significantly upregulated in patients with LN when compared with SLE patients without renal involvement [35], and in LN patients its expression correlates with disease activity [45].

Three lncRNAs (lnc-DC, lnc5150, and lnc7514) individually and as a panel may be used to distinguish SLE with LN from SLE without LN [37].

Low levels of linc0949 significantly correlate positively with C3 levels [44] and also negatively with the SLEDAI-2K score [35,44]. Downregulation of this lncRNA is associated with the presence of cumulative organ damage in patients with SLE and may identify patients with active and severe LN [44]. SLE patients seropositive for anti-dsDNA have a significantly lower level of linc0949 than patients without anti-dsDNA antibodies [35].

Metastasis-associated lung adenocarcinoma transcript 1 (MALAT-1), also known as NEAT2, has upregulated expression in SLE and LN patients [46,45]. MALAT-1 negatively correlates with the estimated glomerular filtration rate (eGFR) in SLE patients without LN. In SLE patients with nephritis MALAT-1 negatively correlates with anti-dsDNA and positively with creatinine levels [45]. It is known for MALAT-1 that it regulates the production of IL-21 in monocytes [46].

CTC-471J1.2 shows high sensitivity and specificity as a diagnostic marker for LN. Expression of CTC-471J1.1 has a negative correlation with SLEDAI scores in all SLE patients and a positive correlation with eGFR in only LN patients [45].

Taurine-upregulated 1 (TUG1) is downregulated lncRNA in LN patients when compared with those patients with SLE alone [47]. Low expression of TUG1 is negatively related to SLEDAI-2K score, ESR, disease duration, and 24-hour urinary protein, and is positively correlated with the level of C3 [47]. Evaluation of TUG1 levels could be a useful biomarker for the diagnosis of SLE and the prediction of complications of LN. Low expression of TUG1 is related to inflammatory injury by regulation of microRNA-223 and SIRT1 levels in SLE patients [34].

CircHLA-C is lncRNA with significantly higher expression in the kidney of SLE patients with class IV LN when compared with healthy individuals [48]. CircHLA-C positively correlates with clinical biomarkers as proteinuria, serum creatinine, renal activity index, and the presence of crescentic glomeruli [48]. The expression levels of circ-HLA-C are not investigated in blood from LN patients but rather on histology.

Another circRNA_002453 is significantly upregulated in LN patients when compared with SLE patients without renal involvement and rheumatoid arthritis patients [49]. These results suggest that circRNA_002453 is a relatively specific biomarker for LN and useful for distinguishing SLE patients with and without LN, as well as its levels positively correlating with proteinuria and renal SLEDAI score [49].

RP11-2B6.2 is upregulated in LN patients, predominantly in those with active LN [50]. It is also found to be elevated in LN patients with active lesions than patients with chronic lesions. Liao et al. report a positive correlation of RP11-2B6.2 with proteinuria and measured interferon (IFN) score [50]. RP11-2B6.2 is regarded as a new positive regulator of the IFN-I signal pathway by inhibition of suppressor of cytokine signaling 1 (SOCS1) in LN [50].

## Conclusions

Since LN is one of the most serious complications of SLE associated with high mortality, establishing new specific biomarkers for the prediction of renal outcome is very important. Investigation of circulating lncRNAs levels in patients could dramatically alter the course of the disease, prolong survival, and improve the quality of life of LN patients. More studies on the significance of expression levels and on the pathogenetic role of lncRNAs in LN are needed. Circulating lncRNAs as next-generation biomarkers have a future as a specific marker with the potential to add to the conventional biomarkers in diagnosis and monitoring of SLE patients and especially those with LN.

## References

[1] Anaya JM, Shoenfeld Y, Rojas-Villarraga A, Levy RA, Cervera R, eds eds (2013). Autoimmunity: From Bench to Bedside. https://pubmed.ncbi.nlm.nih.gov/29087650/.

[2] Schwartz N, Goilav B, Puttermanc C (2014). The pathogenesis, diagnosis and treatment of lupus nephritis. Curr Opin Rheumatol.

[3] Hogan J, Appel GB (2013). Update on the treatment of lupus nephritis. Curr Opin Nephrol Hypertens.

[4] Appel GB, Jayne D (2010). Brenner and Rector's The Kidney. Johnson R, Floege J, Feehaly J.

[5] Zou Y, Xub H (2020). Involvement of long noncoding RNAs in the pathogenesis of autoimmune diseases. J Translat Autoim.

[6] Zhou Z, Sun B, Huang S, Zhao L (2019). Roles of circular RNAs in immune regulation and autoimmune diseases. Cell Death Dis.

[7] Egner W (2000). The use of laboratory tests in the diagnosis of SLE. J Clin Pathol.

[8] Simmons SC, Smith ML, Chang-Miller A, Keddis MT (2015). Antinuclear antibody-negative lupus nephritis with full house nephropathy: a case report and review of the literature. Am J Nephrol.

[9] Qu C, Zhang J, Zhang X, Du J, Su B, Li H (2019). Value of combined detection of anti-nuclear antibody, anti-double-stranded DNA antibody and C3, C4 complements in the clinical diagnosis of systemic lupus erythematosus. Exp Ther Med.

[10] Waldman M, Madaio MP (2005). Pathogenic autoantibodies in lupus nephritis. Lupus.

[11] Hsieh SC, Tsai CY, Yu CL (2016). Potential serum and urine biomarkers in patients with lupus nephritis and the unsolved problems. Open Access Rheumatol.

[12] Linnik MD, Hu JZ, Heilbrunn KR, Strand V, Hurley FL, Joh T; LJP 394 Investigator Consortium (2005). Relationship between anti-double-stranded DNA antibodies and exacerbation of renal disease in patients with systemic lupus erythematosus. Arthritis Rheum.

[13] Adamichou C, Bertsias G (2017). Flares in systemic lupus erythematosus: diagnosis, risk factors and preventive strategies. Mediterr J Rheumatol.

[14] Manson JJ, Ma A, Rogers P (2009). Relationship between anti-dsDNA, anti-nucleosome and anti-alpha-actinin antibodies and markers of renal disease in patients with lupus nephritis: a prospective longitudinal study. Arthritis Res Ther.

[15] Fu SM, Dai C, Zhao Z, Gaskin F (2015). Anti-dsDNA antibodies are one of the many autoantibodies in systemic lupus erythematosus. F1000Res.

[16] Moroni G, Trendelenburg M, Del Papa N (2001). Anti-C1q antibodies may help in diagnosing a renal flare in lupus nephritis. Am J Kidney Dis.

[17] Mok CC, Ho LY, Leung HW, Wong LG (2010). Performance of anti-C1q, antinucleosome, and anti-dsDNA antibodies for detecting concurrent disease activity of systemic lupus erythematosus. Transl Res.

[18] Julkunen H, Ekblom-Kullberg S, Miettinen A (2012). Nonrenal and renal activity of systemic lupus erythematosus: a comparison of two anti-C1q and five anti-dsDNA assays and complement C3 and C4. Rheumatol Int.

[19] Thanei S, Trendelenburg M (2016). Anti-C1q Autoantibodies from systemic lupus erythematosus patients induce a proinflammatory phenotype in macrophages. J Immunol.

[20] Schaller M, Bigler C, Danner D, Ditzel HJ, Trendelenburg M (2009). Autoantibodies against C1q in systemic lupus erythematosus are antigen-driven. J Immunol.

[21] Trendelenburg M, Lopez-Trascasa M, Potlukova E (2006). High prevalence of anti-C1q antibodies in biopsy-proven active lupus nephritis. Nephrol Dial Transplant.

[22] Liu CC, Ahearn JM (2009). The search for lupus biomarkers. Best Pract Res Clin Rheumatol.

[23] Marto N, Bertolaccini ML, Calabuig E, Hughes GR, Khamashta MA (2005). Anti-C1q antibodies in nephritis: correlation between titres and renal disease activity and positive predictive value in systemic lupus erythematosus. Ann Rheum Dis.

[24] Sinico RA, Radice A, Ikehata M (2005). Anti-C1q autoantibodies in lupus nephritis: prevalence and clinical significance. Ann N Y Acad Sci.

[25] Wu FQ, Zhao Q, Cui XD, Zhang W (2011). C1q and anti-C1q antibody levels are correlated with disease severity in Chinese pediatric systemic lupus erythematosus. Rheumatol Int.

[26] Yin Y, Wu X, Shan G, Zhang X (2012). Diagnostic value of serum anti-C1q antibodies in patients with lupus nephritis: a meta-analysis. Lupus.

[27] Mahler M, van Schaarenburg RA, Trouw LA (2013). Anti-C1q autoantibodies, novel tests, and clinical consequences. Front Immunol.

[28] Vasilev VV, Noe R, Dragon-Durey MA (2015). Functional characterization of autoantibodies against complement component C3 in patients with lupus nephritis. J Biol Chem.

[29] Vasilev VV, Radanova M, Lazarov VJ, Dragon-Durey M, Fremeaux-Bacchi V, Roumenina LT (2019). Autoantibodies against C3b—functional consequences and disease relevance. Front Immunol.

[30] Radanova M, Mihaylova G, Ivanova D, Daugan M, Lazarov V, Roumenina L, Vasilev V (2020). Clinical and functional consequences of anti-properdin autoantibodies in patients with lupus nephritis [Online ahead of print]. Clin Exp Immunol.

[31] Birmingham DJ, Irshaid F, Nagaraja HN (2010). The complex nature of serum C3 and C4 as biomarkers of lupus renal flare. Lupus.

[32] Gerald BA, Radhakrishnan J, D’Agati VD (2012). Systemic Lupus Erythematosus. Brener & Rector’s The Kidney.

[33] Chelliah V, Balaraman V, Ilango S, Ramesh S, Bhaba VK, Shivakumar D (2017). Is renal biopsy always necessary to start immunosuppressive therapy in lupus nephritis?. Indian J Rheumatol.

[34] Zhao CN, Mao YM, Liu LN, Li XM, Wang DG, Pan HF (2018). Emerging role of lncRNAs in systemic lupus erythematosus. Biomed Pharmacother.

[35] Wu GC, Li J, Leng RX (2017). Identification of long non-coding RNAs GAS5, linc0597 and lnc-DC in plasma as novel biomarkers for systemic lupus erythematosus. Oncotarget.

[36] Li J, Wu GC, Zhang TP (2017). Association of long noncoding RNAs expression levels and their gene polymorphisms with systemic lupus erythematosus. Sci Rep.

[37] Wu GC, Hu Y, Guan SY, Ye DQ, Pan HF (2019). Differential plasma expression profiles of long non-coding RNAs reveal potential biomarkers for systemic lupus erythematosus. Biomolecules.

[38] Suo QF, Sheng J, Qiang FY, Tang ZS, Yang YY (2018). Association of long non-coding RNA GAS5 and miR-21 levels in CD4+T cells with clinical features of systemic lupus erythematosus. Exp Ther Med.

[39] Zhang F, Wu L, Qian J (2016). Identification of the long noncoding RNA NEAT1 as a novel inflammatory regulator acting through MAPK pathway in human lupus. J Autoimmun.

[40] Wang Y, Chen S, Chen S (2018). Long noncoding RNA expression profile and association with SLEDAI score in monocyte-derived dendritic cells from patients with systematic lupus erythematosus. Arthritis Res Ther.

[41] Wang X, Zhang C, Wu Z, Chen Y, Shi W (2018). CircIBTK inhibits DNA demethylation and activation of AKT signaling pathway via miR-29b in peripheral blood mononuclear cells in systemic lupus erythematosus. Arthritis Res Ther.

[42] Li LJ, Zhao W, Tao SS (2017). Comprehensive long non-coding RNA expression profiling reveals their potential roles in systemic lupus erythematosus. Cell Immunol.

[43] Wang JB, Li J, Zhang TP (2019). Expression of several long noncoding RNAs in peripheral blood mononuclear cells of patients with systemic lupus erythematosus. Adv Med Sci.

[44] Wu Y, Zhang F, Ma J (2015). Association of large intergenic noncoding RNA expression with disease activity and organ damage in systemic lupus erythematosus. Arthritis Res Ther.

[45] Saleh AA, Kasem HE, Zahran E, El-Hefnawy SM (2020). Dysregulation of cell-free long non-coding RNAs (NEAT2, CTC-471J1.2 and lnc-DC) in Egyptian systemic lupus and lupus nephritis patients. Meta Gene.

[46] Yang H, Liang N, Wang M (2017). Long noncoding RNA MALAT-1 is a novel inflammatory regulator in human systemic lupus erythematosus. Oncotarget.

[47] Cao HY, Li D, Wang YP, Lu HX, Sun J, Li HB (2020). Clinical significance of reduced expression of lncRNA TUG1 in the peripheral blood of systemic lupus erythematosus patients. Int J Rheum Dis.

[48] Luan J, Jiao C, Kong W (2018, 2). circHLA-C plays an important role in lupus nephritis by sponging miR-150. Mol Ther Nucleic Acids.

[49] Ouyang Q, Huang Q, Jiang Z, Zhao J, Shi GP, Yang M (2018). Using plasma circRNA_002453 as a novel biomarker in the diagnosis of lupus nephritis. Mol Immunol.

[50] Liao Z, Ye Z, Xue Z (2019). Identification of renal long non-coding RNA RP11-2B6.2 as a positive regulator of type I interferon signaling pathway in lupus nephritis. Front Immunol.

